# Unraveling the relevance of the polyadenylation factor EhCFIm25 in *Entamoeba histolytica* through proteomic analysis

**DOI:** 10.1002/2211-5463.13287

**Published:** 2021-09-13

**Authors:** América Itzallana Salgado‐Martínez, Rodolfo Gamaliel Avila‐Bonilla, Esther Ramírez‐Moreno, Carlos Alberto Castañón‐Sánchez, César López‐Camarillo, Laurence A. Marchat

**Affiliations:** ^1^ Laboratorio de Biomedicina Molecular II ENMH Instituto Politécnico Nacional Mexico City Mexico; ^2^ Hospital Regional de Alta Especialidad de Oaxaca Mexico; ^3^ Posgrado en Ciencias Genómicas Universidad Autónoma de la Ciudad de México (UACM) Mexico

**Keywords:** CFIm25 silencing, *Entamoeba histolytica*, polyadenylation, proteomics, virulence

## Abstract

We recently reported that silencing of the polyadenylation factor EhCFIm25 in *Entamoeba histolytica*, the protozoan which causes human amoebiasis, affects trophozoite proliferation, death, and virulence, suggesting that EhCFIm25 may have potential as a new biochemical target. Here, we performed a shotgun proteomic analysis to identify modulated proteins that could explain this phenotype. Data are available via ProteomeXchange with identifier PXD027784. Our results revealed changes in the abundance of 75 proteins. Interestingly, STRING analysis, functional GO‐term annotations, KEGG analyses, and literature review showed that modulated proteins are mainly related to glycolysis and carbon metabolism, cytoskeleton dynamics, and parasite virulence, as well as gene expression and protein modifications. Further studies are needed to confirm the hypotheses emerging from this proteomic analysis, to thereby acquire a comprehensive view of the molecular mechanisms involved.

## Graphical abstract

The absence of the polyadenylation factor EhCFIm25 affects proliferation, death, and virulence of *Entamoeba histolytica*, the protozoan which causes human amoebiasis. A shotgun proteomic study followed by bioinformatic analysis revealed that these effects are due to changes in the abundance of proteins mainly related to energy metabolism, cytoskeleton dynamics, and gene expression.

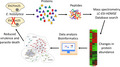

**Abbreviations**
3′ UTR: 3′ untranslated region
ABP: actin‐binding protein
ACD: acetyl‐CoA synthetase_ putative
ADHE: aldehyde‐alcohol dehydrogenase
ALDO: aldolase
CAP: cyclase‐associated protein
CF: cleavage factor
CPBF: cysteine protease‐binding protein family
CPSF: cleavage and polyadenylation specificity factor
CstF: cleavage stimulation factor
dsRNA: double‐stranded RNA
ENO: enolase_ putative
G3PDH: glycerol‐3‐phosphate dehydrogenase
GAPDH: glyceraldehyde 3‐phosphate dehydrogenase
GPP/GK: glycerol‐3‐phosphate phosphatase/glycerol kinase
HK: hexokinase
HPI: phosphoglucose isomerase
MDH: malate dehydrogenase
ME: malic enzyme
PABP: poly(A) binding protein
PAP: poly(A) polymerase
PEPCK3: phosphoenolpyruvate carboxykinase
PFKm: phosphofructokinase
PFOR: pyruvate: ferredoxin oxidoreductase
PGAM: phosphoglycerate mutase
PGK: phosphoglycerate kinase
PK/PPDK: pyruvate_ phosphate dikinase
PPi‐PFK/PFK: pyrophosphate‐fructose 6‐phosphate 1‐phosphotransferase
RNA Pol II: RNA polymerase II
SOD: superoxide dismutase
TPI: triosephosphate isomerase

Gene expression regulation is a key event for eukaryotic cell biology and survival, allowing organisms to adapt to stress, extracellular stimuli, and cell–cell communication by adjusting protein synthesis. After gene transcription by RNA polymerase II (RNA Pol II) in the nucleus, pre‐mRNA molecules are modified by 5′ end capping, splicing, and 3′‐end polyadenylation to generate mature transcripts that travel to cytoplasm to be translated to proteins. These different regulatory steps in gene expression have been studied in *Entamoeba histolytica*, the protozoan parasite responsible for amebic dysentery and amebic liver abscess that represents one of the major leading causes of death from parasitic diseases worldwide, mainly in developing countries [1]. Relevant DNA motifs in promoters and several transcription factors have been reported; capping has not been described yet, but most of the main components of splicing and polyadenylation machineries found in higher eukaryotic cells, have been identified [2]. Our group reported that *E. histolytica* presents the six subunits of the cleavage and polyadenylation specificity factor (CPSF160, CPSF100, CPSF73, CPSF30, Fip1, and WDR33), but only two of the three subunits of the cleavage stimulation factor (CstF77 and CstF64); it has both CLP1 and PCF11 subunits of the cleavage factor IIm (CFIIm), but only the small 25 kDa subunit of the CFIm; the poly(A) polymerase (PAP), the poly(A) binding protein (PABP), RBPP6, and PP1a were also found [3].

In humans, CFImI is a heterotetramer complex with two 25 kDa subunits bound to a dimer of larger subunits (59 or 68 kDa). Notably, CFIm25 is essential for polyadenylation factor recruitment, poly(A) site selection, pre‐mRNA cleavage, and poly(A) tail synthesis [4, 5, 6, 7]. The fact that the 25 kDa subunit is the only CFIm component identified in *E. histolytica* suggests that mRNA polyadenylation could involve a different mechanism in this parasite, and EhCFIm25 could have a central role in this event. Molecular characterization of EhCFIm25 confirmed that it is a Nudix protein although three of the four glutamate residues (E154, E157, and E158) in the conserved Nudix box are replaced by lysine, and the last glycine residue (G160) is substituted by the hydrophilic residue serine. As the human protein, EhCFIm25 interacts with EhPAP [8], but also with the transcriptional coactivator EhPC4 (J. D. Ospina‐Villa, our unpublished data) related to virulence, DNA replication, and multinucleation in *E. histolytica* [9, 10]. Although three‐dimensional structure prediction indicates the absence of a classical RNA‐binding domain, EhCFIm25 can bind the 3′ untranslated region (3′ UTR) of *E. histolytica* transcripts through the participation of the conserved Leu135 and Tyr236 residues [11, 12]. By using the SELEX strategy, we isolated two RNA aptamers that contain the GUUG motif recognized by EhCFIm25. Importantly, EhCFIm25 sequester by aptamers, rapidly induced parasite death, which confirms that targeting the polyadenylation process, namely EhCFIm25, represents an effective strategy for controlling *E. histolytica* [13]. On the other hand, EhCFIm25 silencing produced a significant acceleration in parasite proliferation and cell death; moreover, cells were larger and multinucleated, and their ability to move and phagocyte erythrocytes was significantly reduced, indicating loss of virulence. Additionally, functional experiments showed that EhCFIm25 controls the selection of the distal poly(A) site in mRNA 3′UTR [14]. However, the relationships between EhCFIm25 inhibition and these phenotypical characteristics remain unknown. Here, we performed a shotgun proteomic analysis to evidence changes in protein expression that can explain the observed phenotype.

## Materials and methods

### Microorganisms

*Entamoeba histolytica* trophozoites (strain HMI:IMSS) were grown in aerobic and axenic conditions at 37 °C in TYI‐S‐33 medium with 20% adult bovine serum, 100 U·mL^−1^ penicillin, and 100 μg·mL^−1^ streptomycin [15]. *Escherichia coli* strain HT115 (rnc14:DTn10) was grown at 37 °C in LB and 2YT broth for plasmid pL4440‐EhCFIm25 maintenance and EhCFIm25‐dsRNA expression, respectively, supplemented with ampicillin (100 mg·mL^−1^) and tetracycline (10 mg·mL^−1^) [14].

### *EhCFIm25* gene silencing

Double‐stranded RNA (dsRNA)‐based *EhCFIm25* gene silencing was performed as described [14]. Briefly, *E. histolytica* trophozoites (5.0 × 10^4^) were grown for 4 days in TYI‐S‐33 complete medium in the presence of *EhCFIm25*‐dsRNA molecules (100 μg·mL^−1^) obtained from *E. coli* HT115 (rnc14:DTn10) cells transformed with the pL4440‐*EhCFIm25* plasmid. Every day, a 10 μL aliquot of culture was taken to determine parasite number in a Neubauer chamber and living cells stained by Trypan blue. Experiments were performed twice in triplicate, and results were expressed as mean ± SD. Trophozoites growing in standard conditions (without dsRNA) and treated with *gfp*‐dsRNA were used as controls. Data corresponding to the *EhCFIm25*‐dsRNA condition were compared with both control conditions using the two‐way ANOVA test. *P* < 0.05 was considered as statistically significant.

### RNA and protein isolation

Total proteins and RNA of *E. histolytica* trophozoites treated with *EhCFIm25*‐dsRNA and control cells (day 4) were obtained by the TRIzol reagent (Invitrogen) according to manufacturer’s instructions. Protein quality and amount were verified by 10% SDS/PAGE and the Bradford method before being used in Mass spectrometry assays. RNA integrity and concentration were assessed by agarose gel electrophoresis and spectrophotometric analysis in a spectrophotometer (NanoReady Touch, Hangzhou Lifereal Biotechnology). RNA was used in Real‐Time qRT‐PCR to assess inhibition of *EhCFIm25* mRNA expression and validate proteomic data as described below.

### Mass spectrometry analysis LC‐ESI‐HDMSE

The volume corresponding to 150 μg total proteins obtained from the three culture conditions was delivered to the Laboratorio Nacional de Servicios Experimentales (LaNSE), CINVESTAV (Mexico), for protein identification and absolute quantitation by mass spectrometry analysis LC‐ESI‐HDMSE as described [16, 17]. Raw files containing MS and MS/MS spectra were deconvoluted, compared, and quantified by proteinlynx global server (PLGS) v3.0.3 software against *E. histolytica* (Strain: ATCC 30459/HM‐1:IMSS, downloaded from UniProt, 7959 protein sequences, 3 February 2021) concatenated with reverse database. Workflow parameters were included as described [17]. [All protein identifications reported had a percentage of ≥ 95% reliability (protein autocurate green)]. Full data are included in Table S1.

### Data analysis and bioinformatics

Protein amounts (fmol) in trophozoites treated with *EhCFIm25*‐dsRNA were compared with trophozoites treated with *gfp*‐dsRNA and nontreated control cells; all data were expressed as a base 2 logarithm [18]. Protein abundance changes were used to construct a hierarchical clustering (Euclidean distance) by the heatmapper program (http://www.heatmapper.ca). Proteins displaying at least ± 1.0 absolute fold change in both comparisons, *EhCFIm25*‐dsRNA vs. *gfp*‐dsRNA and *EhCFIm25*‐dsRNA vs. nontreated cells, were considered as differentially expressed and selected for further analyses; proteins that were detected in control cells but not in the *EhCFIm25*‐dsRNA condition were also considered in the study. Selected proteins were used Gene Ontology (GO) enrichment analysis. The GO terms and biochemical pathways of proteins were obtained using DAVID v6.8 (https://david.ncifcrf.gov/) and corroborated in AmoebaDB database (https://amoebadb.org/amoeba/app/), UniProt database (https://www.uniprot.org/), and KEGG PATHWAY database (https://www.genome.jp/kegg/pathway.html). All data were visualized using the CytoScape tools software (https://cytoscape.org/). Additionally, modulated proteins were submitted to STRING analysis using the MCL clustering tool (https://string‐db.org/).

### Real‐Time qRT‐PCR (Real‐Time Quantitative Reverse Transcription‐PCR)

Total RNA was used to synthesize cDNA using the SuperScript III Reverse Transcriptase (Invitrogen) according to manufacturer’s instructions in a GeneQ Thermal Cycler (BIOER, Hangzhou, China). The qPCR assay was completed using SensiFAST™ SYBR Hi‐ROX (Bioline) with specific primers for selected genes as follows: EhCFIm25: sense 5′‐TGGAGAAGATGATCCTGTTGAAG‐3′ and antisense 5′‐TCTTTGACTTGACTTACATGAACTG‐3′ primers [14]; pyruvate phosphate dikinase (PPDK): sense 5′‐CAGCTACTGGTGTTTGTTTCAC‐3′ and antisense 5′‐GATCTGCATCTTCTGCCATCT‐3′ primers; pyruvate:ferredoxin oxidoreductase (PFOR): sense 5′‐CCCAATTACACCATCATCACC‐3′ and antisense 5′‐ATGCTCCAGCTTCACTTTCC‐3′ primers; peroxiredoxin, putative (PRX): sense 5′‐AGCATGGTGTGAAGCAGATAA‐3′ and antisense 5′‐CCTGCTTCGACATTTAACATTCC‐3′ primers; acetyl‐CoA synthetase, putative (ACD): sense 5′‐ACAGAGGAATGCCAGCTTGT‐3′ and antisense 5′‐GGTTGGATGACGAGGTGAG‐3′ primers; actin‐like protein, putative (ARP2/3): sense 5′‐TTCCCAACAGCCATCTTTCCA‐3′‐ and antisense 5′‐GCAGCTGCTTCATCTCCAAAC‐3′ primers; myosin heavy chain: sense 5′‐TGGGTAAAGCTGGAGCACAT‐3′ and antisense 5′‐GTGTCCATGGGATACCTTCGT‐3′ primers; ubiquitin putative: sense 5′‐AGGAATTCCACCTGATCAACAA‐3′ and antisense 5′‐TCTGAAAGTGTCTTTCCTTCTTCT‐3′ primers; PC4: sense 5′‐AAAACTTCCATTTGACGGTGACAA‐3′ and antisense 5′‐TCCTGGTTTTA ATTCTCCATCTCT‐3′ primers; and PAP: sense 5′‐GTGCAGGAGTTGCTGATGAC‐3′ and antisense 5′‐TGTGGTGATCGTTTTGATGGA‐3′primers. The EhRNAPII gene was used for normalization (sense 5′‐GATCCAACATATCCTAAAACAACA‐3′ and antisense 5′‐TCAATTATTTTCTGACCCGTCTTC‐3′ primers) [19]. All reactions were performed in a StepOne real‐time PCR system (Applied Biosystems) with the following cycling conditions: enzyme activation at 95 °C for 2 min, followed by 40 cycles of denaturation at 95 °C for 5 s and annealing/extension at 62 °C for 30 s. The experiment was carried out by triplicated, and the relative expression of mRNA was determined by the 2^−ΔΔCt^ method. Data corresponding to the *EhCFIm25*‐dsRNA condition were analyzed by Prism GraphPad software using paired Student’s *t*‐test to compare the *EhCFIm25*‐dsRNA condition with both control conditions. *P* < 0.05 was considered as statistically significant.

## Results

### Silencing of *EhCFIm25* by dsRNA

To evidence how the absence of EhCFIm25 affects protein expression, we first induced downregulation of *EhCFIm25* gene expression by soaking trophozoites with purified *EhCFIm25*‐dsRNA as described [14]. As expected, real‐time qRT‐PCR assays confirmed that on day 4, *EhCFIm25* gene transcription was reduced by about 75% in parasite cultures containing *EhCFIm25*‐dsRNA, when compared with *gfp*‐dsRNA‐treated cells and nontreated cells. Additionally, trophozoites exhibited an accelerated proliferation from day 2 when compared with both control cells, as well as a higher number of dead cells (Fig. S1). All these observations agreed with our previous report [14] and strongly supported the downregulation of *EhCFIm25* expression following *EhCFIm25*‐dsRNA soaking.

### Identification of modulated proteins following *EhCFIm25* silencing

**Fig. 1:**
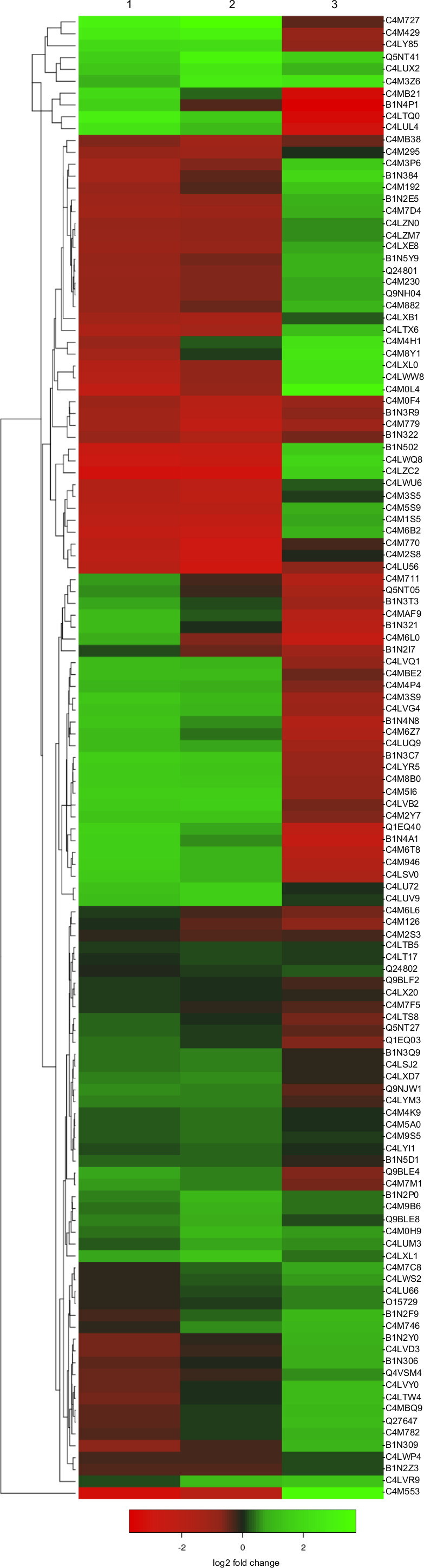
Protein abundance changes in *E. histolytica* trophozoites treated with *EhCFIm25*‐dsRNA and controls. The heat map shows the hierarchical clustering (Euclidean distance) of base 2 logarithm protein abundance. Lane 1, *EhCFIm25*‐dsRNA‐treated cells vs. *gfp*‐dsRNA condition; lane 2, *EhCFIm25*‐dsRNA‐treated cells vs. and nontreated cells; lane 3, *gfp*‐dsRNA‐treated cells vs. nontreated cells. Color key: increased proteins (green); reduced proteins (red). The 15 proteins that were no detected in *EmCFIm25* silenced cells are not represented here.

Protein extracts were obtained from *EhCFIm25*‐dsRNA‐treated trophozoites and both control cultures (*gfp*‐dsRNA condition and nontreated cells) and analyzed by mass spectrometry analysis LC‐ESI‐HDMSE as described [16, 17, 18]. A total of 139 proteins (including 11 uncharacterized proteins) was identified and quantified, considering 99.9% accuracy and detection of at least two peptides for protein identification (Table S1). Hierarchical clustering evidenced that many proteins showed similar changes in abundance when comparing *EhCFIm25*‐dsRNA‐treated cells vs. *gfp*‐dsRNA condition and nontreated cells, indicating that *EhCFIm25* silencing had an effect on the gene expression (Fig. 1). Because of the relevance of EhCFIm25 in polyadenylation and the role of poly(A) tail in translation and mRNA stability, the absence of EhCFIm25 probably affected mRNA turnover and translation; however, we cannot discard that the turnover of some proteins could also be changed.

**Fig. 2:**
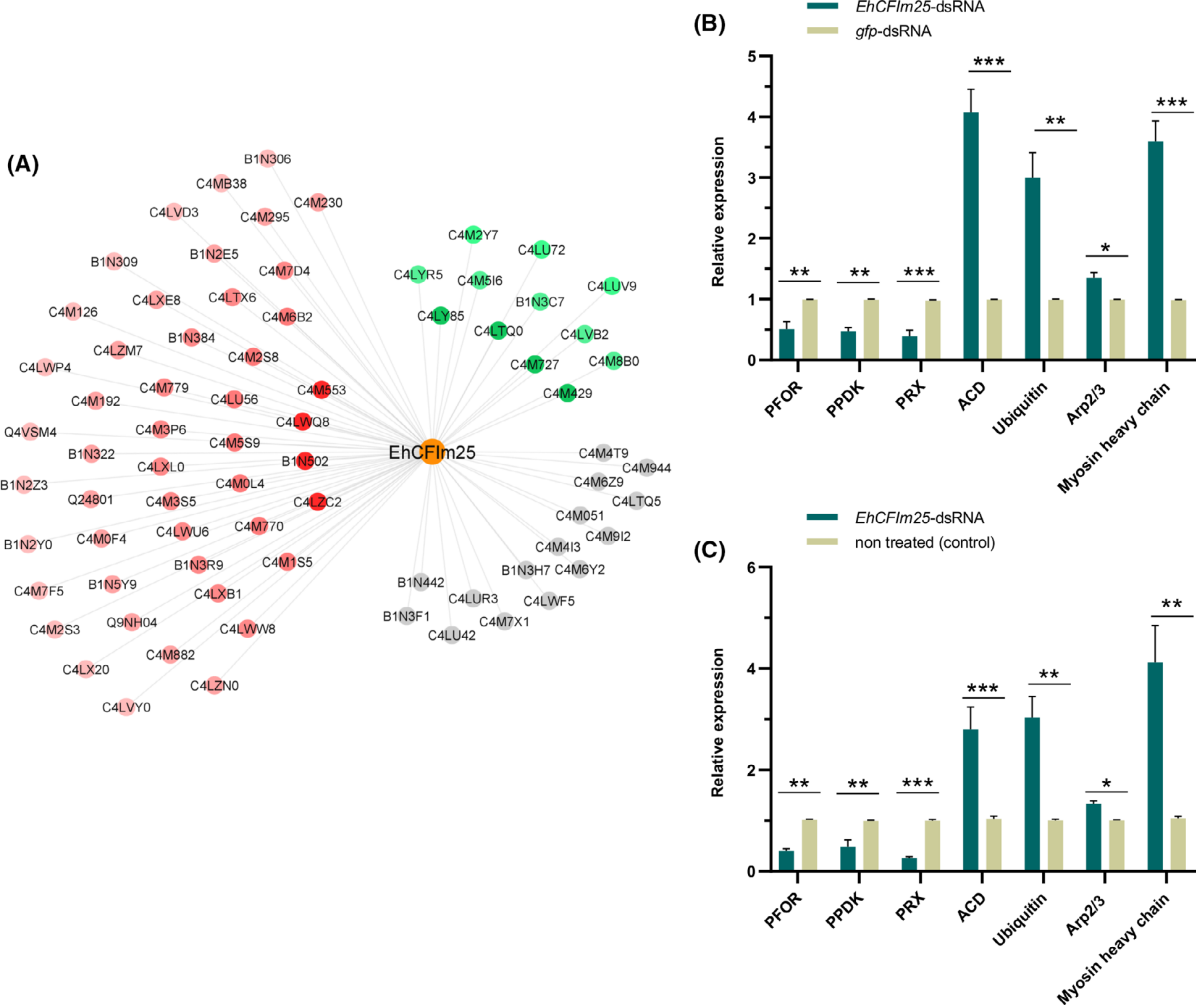
*E. histolytica* proteins modulated in response to *EhCFIm25* silencing. (A) Schematic representation of proteins with significant changes in abundance following *EhCFIm25* silencing. Red, proteins in a reduced amount; green, proteins in a higher amount; gray, proteins that were not detected. The color intensity is proportional to log2 fold change value. (B and C) Real‐time quantitative reverse transcription polymerase chain reaction (real‐time qRT‐PCR) for seven selected genes in *EhCFIm25*‐dsRNA trophozoites compared with (A) nontreated cells and (C) *gfp*‐dsRNA‐treated parasites. The *EhRNAPII* gene mRNA expression was determined and used as normalization control for all qRT‐PCR assays. Data corresponding to the *EhCFIm25*‐dsRNA condition were compared with both control conditions using the paired Student's *t*‐test. **P < 0.01; ***P < 0.001. *n* = 3. Error bars represent SD.

Of these 139 proteins, 75 showed a significant log2 fold change value when comparing *EhCFIm25*‐dsRNA‐treated cells vs. both control conditions. These included 48 less abundant proteins (comprising four uncharacterized proteins) and 12 more abundant proteins (with two uncharacterized proteins); notably, 15 proteins (including four uncharacterized proteins) were not detected in the absence of EhCFIm25 (Fig. 2A). The four most reduced proteins were the PPi‐type phosphoenolpyruvate carboxykinase 3 (C4LWQ8) (PPi‐PEPCK3/PEPCK3), the pyrophosphate‐fructose 6‐phosphate 1‐phosphotransferase (C4LZC2) (PPi‐PFK/PFK), and the 3‐ketoacyl‐CoA synthase 4 (B1N502) that are related to metabolic pathways, such as glycolysis, and lipid metabolism, respectively, as well as a leucine‐rich (LRR) repeat containing protein (C4M553). On the other hand, three of the highly enriched proteins corresponded to the 60S ribosomal protein L14 (C4LTQ0), 60S ribosomal protein L27 (C4M727), and ubiquitin (C4LY85), indicating that *EhCFIm25* silencing had an impact on protein synthesis. The fourth one is an uncharacterized protein (C4M429). To validate changes in protein abundance, we selected genes corresponding to three proteins with a reduced abundance, PFOR, PPDK, and PRX, and four proteins with an increased amount, ACD, ARP2/3, myosin heavy chain ubiquitin, and evaluated mRNA expression by real‐time RT‐qPCR in the three groups of trophozoites. In agreement with proteomics data, PFOR, PPDK, and PRX genes were downregulated in response to *EhCFIm25* silencing in comparison with the untreated cells and the *gfp*‐dsRNA condition, while ACD, ARP2/3, and myosin heavy chain ubiquitin genes were upregulated (Fig. 2B,C). We previously showed that EhCFIm25 interacts with EhPAP and EhPC4, two proteins involved in polyadenylation and transcription, respectively. Although these nuclear proteins did not appear in the proteomes, we investigate whether there were any changes in the mRNA expression of EhPAP and EhPC4 genes upon silencing the EhCFIm25. Results showed mRNA expression of both genes was increased in EhCFIm25‐silenced trophozoites (Fig. S2).

**Fig. 3:**
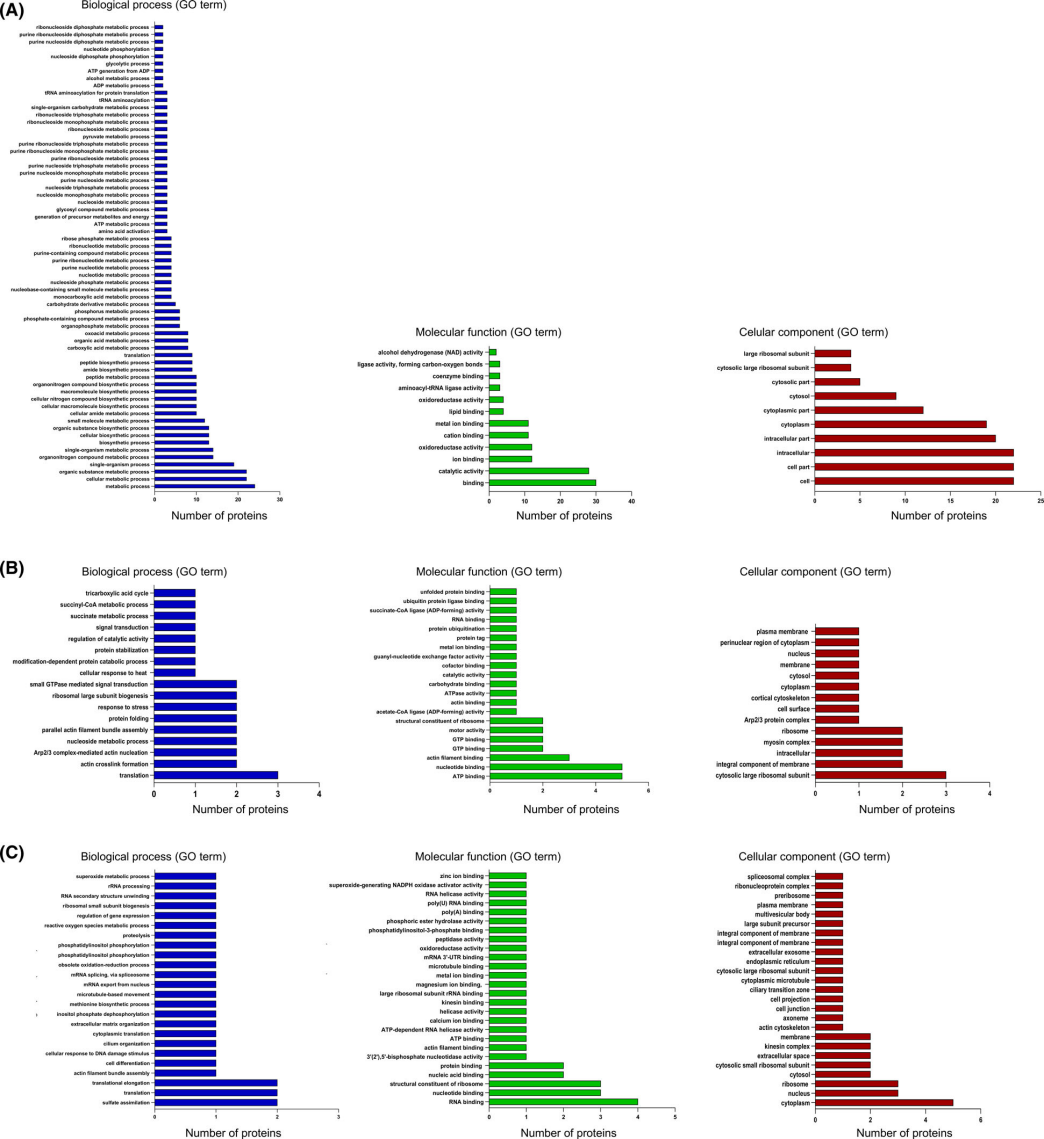
Functional categorization of proteins with modulated abundance following *EhCFIm25* silencing. Proteins with a reduced abundance (A), an increased abundance (B), and proteins that were not detected (C) were classified according to cellular component, biological process, and molecular function, defined from Gene Ontology categories by DAVID.

With the aim of having a general view of the impact of change in protein abundance in *EhCFIm25*‐silenced trophozoites, the identified proteins were categorized using functional GO‐term annotations. The 10 uncharacterized proteins were not included since BLAST analyses failed to assign them a possible identity and function (data not shown). Many of the less abundant proteins corresponded to cellular and intracellular compartments; concerning biological process, they were mainly associated with metabolism, including metabolic process, organic substance metabolic process, and cellular metabolic process; finally, the main function was binding. Regarding proteins that were more abundant in the absence of EhCFIm25, they were mainly found as part of the ribosome and cytoplasm, and mostly related to Arp2/3 complex‐mediated actin nucleation and translation; interestingly, the main molecular functions were related to binding, including RNA, ATP, and nucleotide binding, as well as ATPase activity. Lastly, proteins that were not detected in *EhCFIm25*‐silenced trophozoites corresponded to cytosol and ribosome; they were mainly associated with translation and related to nucleotide and RNA binding, as well as part of the structural constituent of ribosome (Fig. 3).

**Fig. 4:**
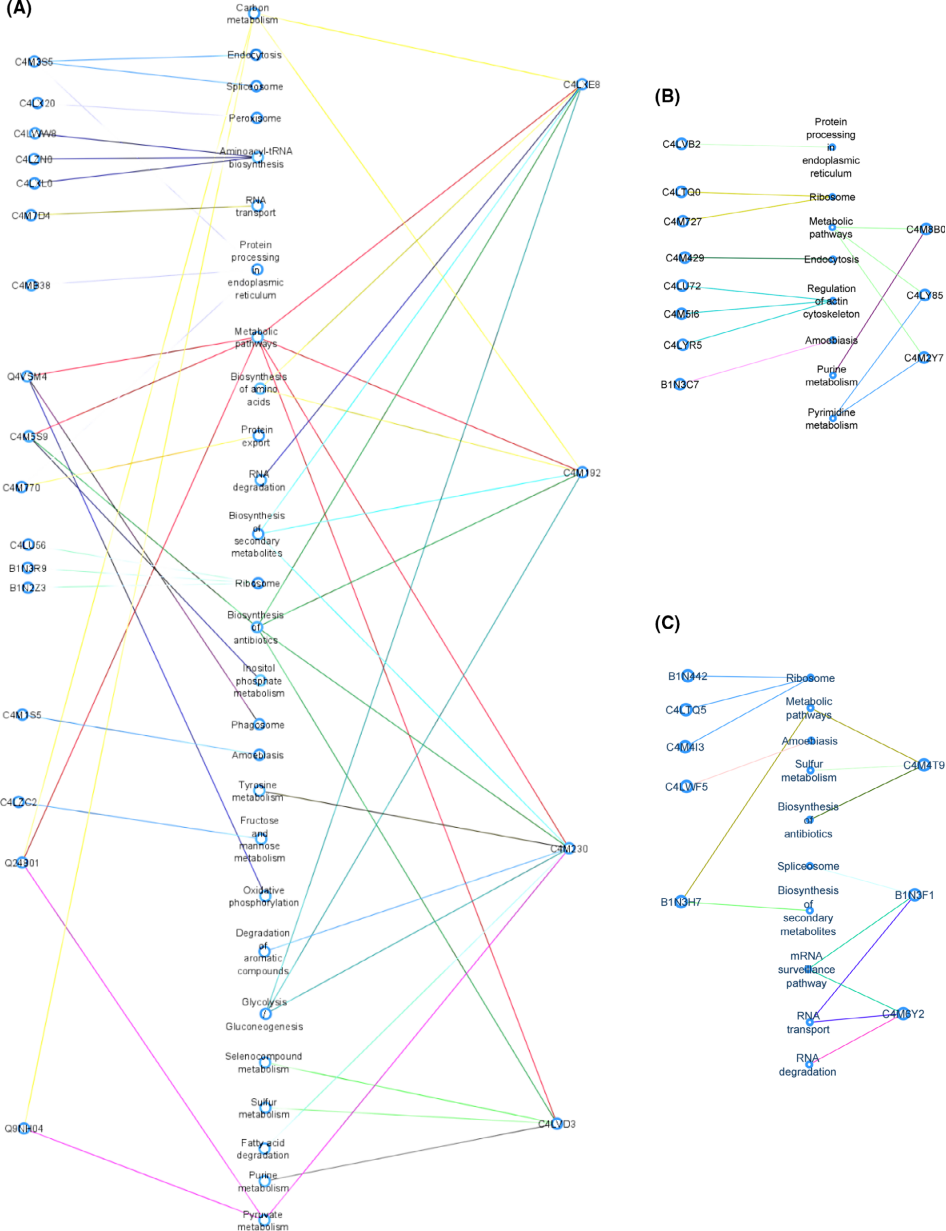
KEGG pathways associated with proteins with a reduced abundance (A), an increased abundance (B), and proteins that were not detected (C) following *EhCFIm25* silencing.

KEGG analyses evidenced that four of the proteins with a reduced abundance, C4LXE8, C4M192, C4M230, and C4LVD3, have an impact on at least five biochemical pathways. They also confirmed that reduced proteins are mainly related to metabolism, namely carbon metabolism, metabolic pathways, glycolysis/gluconeogenesis, and pyruvate metabolism; they were also related to gene expression regulation by acting in spliceosome, protein processing and endoplasmic reticulum, ribosome, RNA transport, aminoacyl‐t RNA biosynthesis, and biosynthesis of amino acids. Regarding proteins with an increased abundance, three proteins (C4M8B0, C4M2Y7, and C4LY85) were involved in two biochemical pathways. Regulation of actin cytoskeleton was the most affected pathway. On the other hand, three of the proteins that were not detected in *EhCFIm25*‐silenced trophozoites (C4M4T9, B1N3F1, and C4M6Y2) participate in three biochemical pathways. Gene expression regulation, including RNA transport and mRNA surveillance, spliceosome, and ribosome, was the most affected process (Fig. 4).

**Fig. 5:**
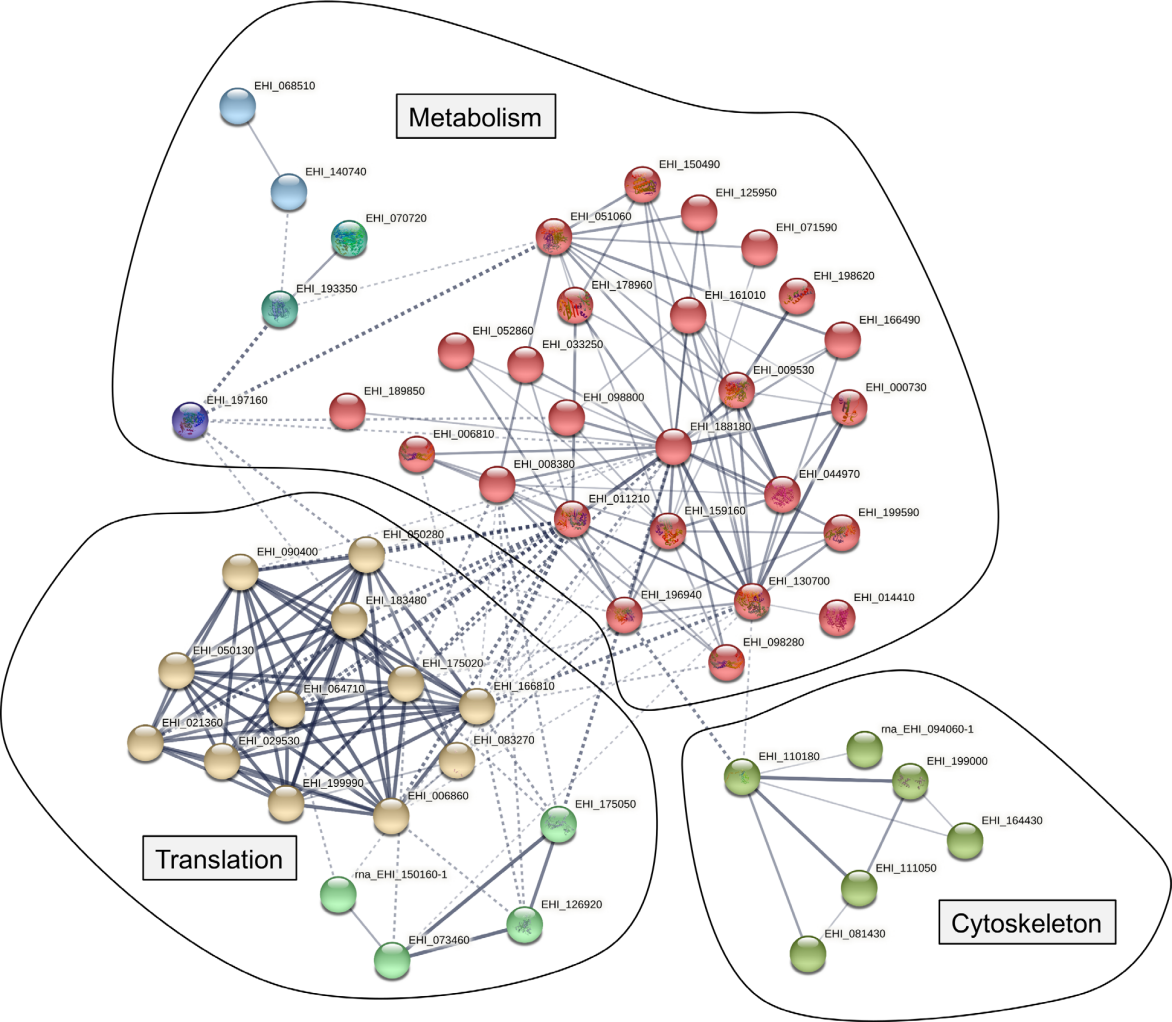
Protein–protein interaction network visualized by STRING. Colored nodes correspond to query proteins and first shell of interactions. The color saturation of the edges represents the confidence score of the association between modulated proteins

In order to better understand how *EhCFIm25* silencing affected *E. histolytica* trophozoites, these functional categorization analyses were completed by a STRING analysis. Interestingly, modulated proteins formed three main groups that remain interconnected through several proteins. One group correspond proteins related to metabolism including glycolysis (EHI_00070, EHI_188180, EHI_130700, EHI_009530, 051060, EHI_178960, EHI_150490), 14‐3‐3 proteins (EHI_098280, EHI_006810), among others, as well as heat‐shock proteins (EHI_199590, EHI_052860; EHI_196940); the other corresponds to cytoskeletal proteins (EHI_110180, EHI_111050, EHI_094060, EHI_154430, and EHI_199000), and the last one is related to translation with the presence of ribosomal proteins (e.g., EHI_050130, EHI_183480, EHI_006860), aminoacyl‐tRNA synthetases (such as EHI_126920 and EHI_073460), elongation factors (EHI_166810, EHI_ EHI_011210), and among others (Fig. 5). Finally, manual annotation of *E. histolytica* proteins and retrieving of published literature allowed us to cluster modulated proteins into three large biological processes, that is, glycolysis and carbon metabolism, cytoskeleton dynamics and parasite virulence, and gene expression and protein modifications (Table 1).

**Table 1:** Modulated proteins in *EhCFIm25*‐silenced trophozoites

Gene ID	UniProt	NCBI	Protein description	*EhCFIm25‐*dsRNA vs nontreated (control) (log2 fold change)	*EhCFIm25‐*dsRNA vs *gfp*‐dsRNA (log2 fold change)	*gfp*‐dsRNA vs nontreated (control) (log2 fold change)
Glycolysis and carbon metabolism						
Increased						
EHI_178960	C4LUV9	XP_656290.1	ACD	1.571976609	1.838014699	0.26603809
Reduced						
EHI_000730	C4LZC2	XP_653173.1	PPi_PFK	−8.953922033	−6.824743753	2.129178281
EHI_188180	C4M192	XP_653686.1	PGK	−3.53422426	−1.70492415	1.82930011
EHI_130700	C4LXE8	XP_649161.1	ENO	−3.664724348	−2.623408105	1.041316242
EHI_051060	C4LTX6	XP_657019.2	PFOR	−5.023063656	−3.386220638	1.636843018
EHI_009530	Q24801	XP_657332.1	PK/PPDK	−3.443328065	−2.212267448	1.231060617
EHI_150490	C4M230	XP_652300.1	ADHE	−3.338295165	−2.276880037	1.061415128
EHI_125950	C4LVY0	XP_650419.1	Alcohol dehydrogenase_putative (ADH)	−2.493758657	−1.026187504	1.467571153
EHI_166490	C4M7F5	XP_652262.2	Alcohol dehydrogenase_putative (ADH)	−1.048881287	−1.286563624	−0.237682337
EHI_198620	C4LWQ8	XP_655201.2	PPi‐PEPCK3	−8.318126762	−5.902664315	2.415462446
EHI_044970	Q9NH04	XP_648590.1	ME	−3.371544637	−2.282862579	1.088682059
EHI_014410	B1N2Y0	XP_001913546.1	MDH_ putative	−2.539964263	−1.29009284	1.249871423
EHI_070720	C4M5S9	XP_654310.1	L‐myo‐inositol‐1‐phosphate synthase	−5.862557542	−4.718114024	1.144443518
Not detected						
EHI_161010	B1N3H7	XP_001913743.1	Anaerobic G3PDH subunit A_ putative	‐	‐	2.79771936
Cytoskeleton dynamics and parasite virulence						
Increased						
EHI_110180	C4LU72	XP_657028.1	Myosin heavy chain	1.742008798	1.915355481	0.173346683
EHI_111050	C4M5I6	XP_657596.1	ARP2	2.48265	1.660252011	−0.822397989
EHI_058090	C4M8B0	XP_651163.1	Ras family GTPase	2.231234072	1.425210378	−0.806023694
EHI_196940	C4LVB2	XP_653132.1	Heat‐shock protein 90_ putative	2.326638075	1.816849869	−0.509788206
EHI_133900	B1N3C7	XP_001913693.1	Galactose‐inhibitable lectin 170 kDa subunit_ putative (Gal/GalNAc lectin)	2.50643645	1.484150956	−1.022285494
Reduced						
EHI_105210	C4M126	XP_653931.1	F‐BAR domain‐containing protein	−1.174858759	−1.873880924	−0.699022165
EHI_094060	B1N322	XP_001913588.1	Actin‐binding protein_ putative (filamin)	−3.592858259	−4.086887697	−0.494029438
EHI_164430	C4M1S5	XP_648375.2	Actinin‐like protein_ putative (α‐actinin 1)	−6.424663932	−5.410549507	1.014114425
EHI_199000	C4LWU6	XP_653283.1	Calponin homology domain protein_ putative (α‐actinin 2)	−5.42520244	−4.937989077	0.487213363
EHI_136150	C4M295	XP_655240.2	Adenylyl CAP	−3.26152685	−3.039265215	0.222261635
EHI_177990	C4M553	XP_651490.1	Leucine‐rich repeat containing protein	−9.129552234	−4.675234233	4.454318001
EHI_167060	B1N309	XP_001913575.1	Rab GDP dissociation inhibitor	−2.913843088	−1.601375505	1.312467583
EHI_098280	C4LXB1	XP_653621.1	14‐3‐3 protein (EhP2)	−4.15541176	−3.696594368	0.458817392
EHI_006810	C4M0F4	XP_654465.1	14‐3‐3 protein 2 (EhP3)	−3.628432526	−4.653662911	−1.025230385
EHI_159160	C4LX20	XP_648827.1	SOD	−1.016518349	−1.037119891	−0.020601541
EHI_121620	B1N5Y9	XP_001914605.1	Peroxiredoxin_ putative (PRDX)	−3.343027913	−2.14245569	1.200572222
EHI_156710	B1N502	XP_001914268.1	3‐ketoacyl‐CoA synthase 4_ putative	−7.753320965	−5.739869952	2.013451013
EHI_199590	C4M770	XP_654737.1	70 kDa heat‐shock protein_ putative	−6.306522113	−6.50351067	−0.196988557
EHI_052860	C4M3S5	XP_650458.1	Heat‐shock protein 70_ putative	−5.496325809	−5.150576387	0.345749422
Not detected						
EHI_187770	C4M9I2	XP_656019.2	SH3 domain protein	‐	‐	0.303916225
EHI_096420	C4LWF5	XP_656918.1	LIM zinc finger domain‐containing protein (LimA)	‐	‐	−0.78774608
EHI_178470	C4LUR3	XP_653036.1	CPBP6	‐	‐	−0.611080228
Gene expression and protein modification						
Increased						
EHI_050130	C4LTQ0	XP_657191.1	60S ribosomal protein L14_ putative	5.702363161	1.648304292	−4.054058869
EHI_183480	C4M727	XP_654328.1	60S ribosomal protein L27_ putative	6.085963509	5.75025083	−0.335712679
EHI_166800	C4LY85	XP_654066.2	Ubiquitin_ putative	3.628806975	2.824287894	−0.804519081
Reduced						
EHI_006860	C4LU56	XP_650508.1	60S ribosomal protein L5_ putative	−5.900697969	−6.602091887	−0.701393918
EHI_064710	B1N384	XP_001913650.1	60S ribosomal protein L4_ putative	−4.30857207	−1.809131252	2.499440818
EHI_126920	C4LWW8	XP_651543.1	Asparaginyl‐tRNA synthetase_ putative	−5.641036285	−2.595619356	3.045416929
EHI_073460	C4LXL0	XP_656678.1	Glycyl‐tRNA synthetase_ putative	−5.479050074	−2.509932008	2.969118066
EHI_029530	B1N3R9	XP_001913834.1	60S ribosomal protein L7a_ putative	−4.111168396	−4.816620213	−0.705451818
EHI_175050	C4LZN0	XP_649894.1	Aspartyl‐tRNA synthetase_ putative	−3.438382911	−2.571822649	0.866560262
EHI_166810	B1N306	XP_001913572.1	Elongation factor 2 (EF2)	−2.301951732	−1.132943057	1.169008675
EHI_011210	C4M7D4	XP_651869.1	Elongation factor 1 alpha (EF1‐α)			
EHI_090400	B1N2Z3	XP_001913560.1	60S acidic ribosomal protein P0	−2.115985101	−1.731688644	0.384296458
EHI_008380	C4M0L4	XP_652558.1	Aminopeptidase	−6.89523807	−2.603764161	4.291473908
EHI_153640	B1N2E5	XP_001913371.1	Protein kinase domain‐containing protein	−3.876761502	−2.627397512	1.24936399
EHI_071590	C4MB38	XP_650651.1	Protein disulfide isomerase_ putative	−2.7241992	−3.178404428	−0.454205228
Not detected						
EHI_138770	C4M4I3	XP_650140.2	60S acidic ribosomal protein P2_ putative	‐	‐	−6.131527277
EHI_050280	C4LTQ5	XP_648594.1	40S ribosomal protein S3a	‐	‐	1.176668332
EHI_199990	B1N442	XP_001913958.1	40S ribosomal protein S6	‐	‐	−1.587461998
EHI_150160	B1N3F1	XP_001913717.1	ATP‐dependent RNA helicase DDX39_ putative	‐	‐	0.739544932
EHI_033250	C4M6Y2	XP_650900.1	Polyadenylate‐binding protein_ putative (PABP)	‐	‐	−0.121076556
EHI_193350	C4M4T9	XP_651950.1	3′ (2′)_5′‐bisphosphate nucleotidase_ putative	‐	‐	3.250181479

## Discussion

We previously reported that EhCFIm25 is essential for *E. histolytica* survival and virulence properties, since its silencing affected trophozoite proliferation, produced parasite death, altered cell size and nucleus number, and reduced mobility and erythrophagocytosis capacity of parasites, which prompted us to propose this polyadenylation factor as a new biochemical target in this human pathogen [14]. Our proteomic analysis revealed that the absence of EhCFIm25 produced changes in the abundance of 75 proteins. Among these, we focused on proteins related to glycolysis and carbon metabolism, cytoskeleton dynamics and parasite virulence, and gene expression and protein modifications.

### *EhCFIm25* silencing affects energy metabolism causing parasite death

**Fig. 6:**
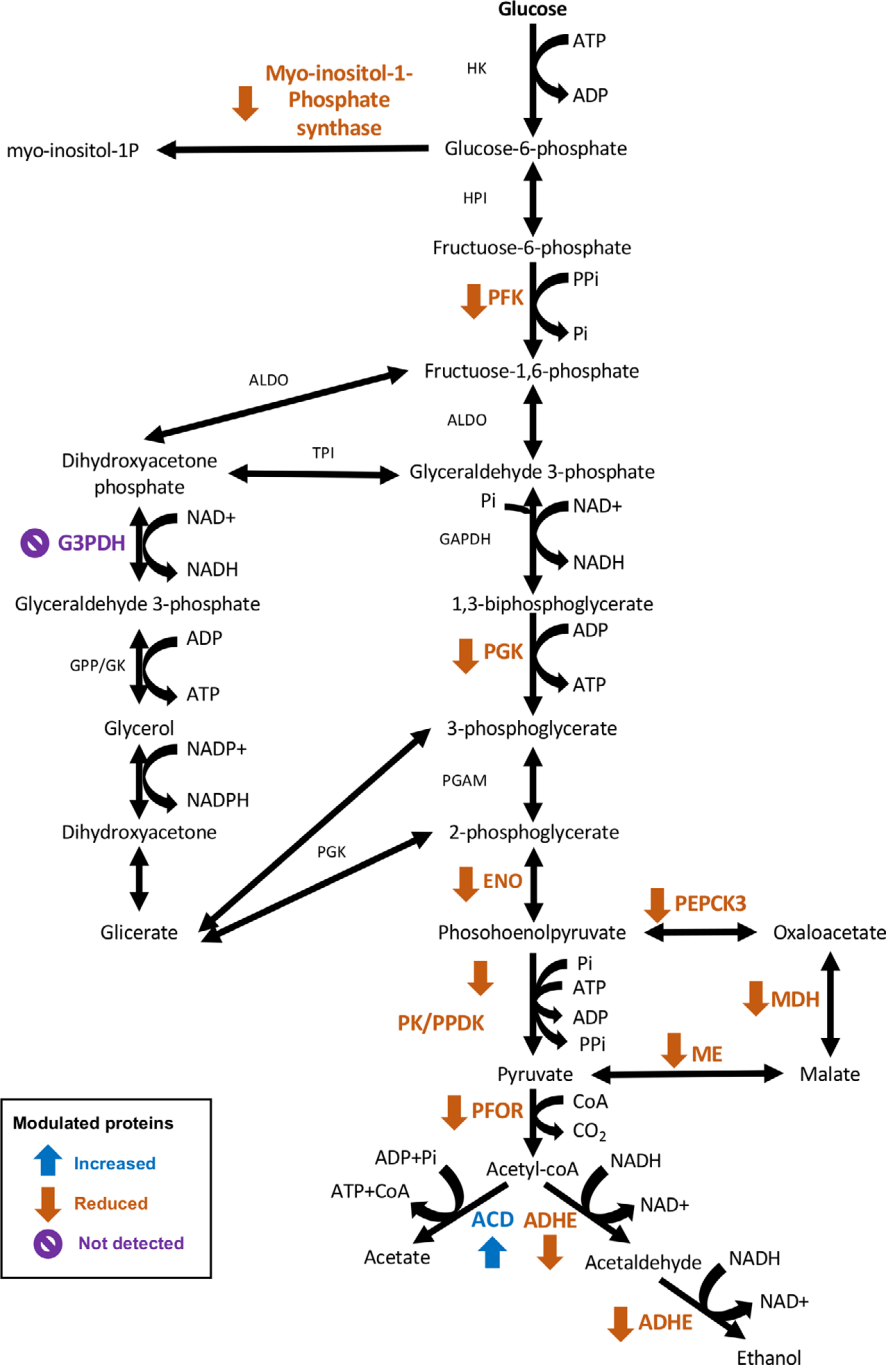
Representation of protein changes in the atypical glycolysis of *E. histolytica* and related pathways in EhCFIm25‐silenced trophozoites. Abbreviations are as follows: HK, hexokinase; HPI, phosphoglucose isomerase; PFKm phosphofructokinase; ALDO, aldolase; GAPDH, glyceraldehyde 3‐phosphate dehydrogenase; PGK, phosphoglycerate kinase; PGAM, phosphoglycerate mutase; ENO, enolase; PK/PPDK, pyruvate, phosphate dikinase; PFOR, pyruvate: ferredoxin oxidoreductase; ADHE, aldehyde‐alcohol dehydrogenase; ACD, acetyl‐CoA synthetase (ADP forming); PEPCK3, phosphoenolpyruvate carboxykinase; MDH, malate dehydrogenase; ME, malic enzyme; TPI, triosephosphate isomerase; G3PDH, glycerol‐3‐phosphate dehydrogenase; GPP/GK, glycerol‐3‐phosphate phosphatase/glycerol kinase.

*E. histolytica* trophozoites lack a functional Krebs cycle and oxidative phosphorylation enzymes and exclusively depend on an unusual PPi‐dependent glycolytic pathway for ATP generation from glucose fermentation. The acetyl‐CoA molecule obtained from the pyruvate end product of glycolysis can be transformed to ethanol by the aldehyde‐alcohol dehydrogenase (ADHE also known as ADH2) in a two‐step reaction or acetate by the ADP‐forming ACD [20]. Our results evidenced the reduced abundance of PPi_PFK/PFK (C4LZC2), phosphoglycerate kinase (C4M192) (PGK), enolase_ putative (C4LXE8) (ENO), PK/PPDK (Q24801), PFOR (C4LTX6), and ADHE (C4M230), which results in an overall reduction in ethanol and ATP production. Other glycolysis‐related enzymes were also affected, such as PPi‐type phosphoenolpyruvate carboxykinase 3 (C4LWQ8) (PPi‐PEPCK3), malic enzyme (Q9NH04) (ME), and malate dehydrogenase_ putative (B1N2Y0) (MDH) that control the PEP‐oxaloacetate‐malate‐pyruvate cycle, the L‐myo‐inositol‐1‐phosphate synthase (C4M5S9) that catalyzes the conversion of glucose 6‐phosphate to 1‐l‐myo‐inositol‐1‐phosphate, and the anaerobic glycerol‐3‐phosphate dehydrogenase subunit A_ putative (B1N3H7) (G3PDH) that converts glycerol‐3‐phosphate to dihydroxyacetone phosphate [21]. Interestingly, the increased abundance of the acetyl‐CoA synthetase_ putative (C4LUV9) (ACD) and the reduced amount of bifunctional ADHE (C4M230), and C4LVY0 and C4M7F5, two putative alcohol dehydrogenases of 42 kDa and 46 kDa, respectively, that did not correspond to the reported ADH1 (39 kDa) and ADH3 (43 kDa), suggested that parasites direct the last steps of glycolysis to the conversion of acetyl‐CoA into acetate with the production of ATP in an attempt to restore, at least partially, the lack of energy that results from downregulation of upstream glycolytic enzymes (Fig. 6). However, energy deficiency is likely the main cause of parasite death following *EhCFIm25* silencing. In agreement with this assumption, it has been reported that ADHE activity is an important control point for the glycolytic flux and its inhibition can decrease the parasite energy load and survival [22].

### *EhCFIm25* silencing affects cytoskeleton dynamics reducing parasite virulence

The cytoskeleton dynamics is essential for various cellular functions in *E. histolytica*, from cytokinesis to morphogenesis, including adhesion, endocytosis, phagocytosis, motility, and migration, that are pivotal activities in amoebic pathogenesis [23]. Although it is the key cytoskeletal protein, actin dynamics requires a coordinated cooperation with a large number of actin‐binding proteins (ABPs) (including myosin II). Recently, a bioinformatic approach identified a total of 390 ABPs, most of them corresponding to uncharacterized proteins [24]. Here, we found that several proteins linked to the actin‐rich cytoskeleton were modulated in EhCFIm25‐silenced trophozoites. Notably, eight ABPs (C4M126, C4M9I2, B1N322, C4M1S5, C4LW6, C4M295, C4M553, and C4LWF5) were downregulated in *EhCFIm25*‐silenced trophozoites. C4M126 (F‐BAR domain‐containing protein) and C4M9I2 (SH3 domain protein) contain a BAR (bin‐amphiphysin‐Rvs) domain that has been involved in membrane curvature and actin binding. In *E. histolytica*, phagocytosis was inhibited by EhBAR (C4M128) silencing, indicating its participation in actin dynamics [25]. In addition, the SH3 domain is enriched in proteins that are upregulated in phagocytic trophozoites [26]. The atypical filamin (B1N322) [27], α‐actinin 1 (C4M1S5) [28], and α‐actinin 2 (C4LWU6) [29] are members of the calponin family that are able to cross‐link actin to stimulate an active net; filamins, particularly filamin A also known as ABP120, have been localized in pseudopods and uropods [30], while α‐actinin 2 (EhActn2) was involved in phagocytic cup formation [31]. The adenylyl cyclase‐associated protein (C4M295) of the cyclase‐associated protein (CAP) family is involved in actin remodeling [32] and nutritional response signaling [33]. The leucine‐rich repeat containing protein (C4M553), an I/LWEQ domain‐containing protein, has two leucine‐rich repeats that are thought to promote protein interactions with surface proteins [34] and a FERM domain that links cytoskeleton proteins to the plasma membrane [35]. EhLimA (C4LWF5) is a LIM domain‐containing protein that colocalized to the plasma membrane with actin filaments in *E. histolytica* [36]. These alterations in ABP amount can be related to the reduced abundance of a small Ras family GTPase (C4M8B0) and a Rab GDP dissociation inhibitor (B1N309) known to regulate the GDP‐GTP exchange reaction of members of the Rab family, leading to a reduction in vesicular trafficking.

On the other hand, only two cytoskeletal‐related proteins were found with an increased abundance: the heavy chain of myosin II (C4LU72) which binds F‐actin and is responsible for cell contraction and motility [37] and ARP2 (C4M5I6), one of the seven subunits of the ARP2/3 complex that mediates actin nucleation and has been localized in phagosomes [38]. Globally, changes observed in the abundance of these cytoskeletal proteins likely affect cytoskeleton organization and dynamics, which can be related to the augmented size of trophozoites and the increased number of nuclei in *EhCFIm25*‐silenced trophozoites, probably due to failure in cytokinesis; cytoskeleton alterations could also be responsible for the reduced mobility and erythrophagocytosis capacity in trophozoites, affecting their virulence capacity [14].

Congruently with these observations, our results evidenced the downregulation of other proteins previously related to phagocytosis, an essential process for parasite nutrition and pathogenesis. Members of the cysteine protease‐binding protein family (CPBF), a single‐transmembrane carrier/receptor family, interact with lysosomal hydrolytic enzymes and regulate their trafficking; notably, CPBF6 (C4LUR3) was identified in phagosomes, participating in the transport of cargo proteins, namely α‐amylase and γ‐amylase [39]. EhP3 (C4M0F4), a homolog of 14‐3‐3 family of protein, participates in initiation/formation of phagocytic cups and formation of phagosome, by acting as an adaptor molecule to recruit proteins that allow dynamics of F‐actin rearrangement during phagocytosis [40]. Several heat‐shock proteins (HSP) have also been involved in parasite virulence; notably, the activity of HSP70 is necessary for amoebic liver abscess formation in hamsters [41], while HSP90 controls the process of phagocytosis [42]. Then, the modulation of HSP90 (CELVB2) and HSP70 (C4M770, C4M3S5) could contribute to the reduced virulence capacity of *EhCFIm25*‐silenced parasites. In addition to phagocytosis, the virulence process of *E. histolytica* includes adherence to target cells, cytolysis, and evasion of host immune response with the participation of various key proteins [43]. Therefore, the reduced amount of the galactose‐inhibitable lectin 170 kDa subunit (B1N3C7) (Gal/GalNAc lectin) located on the surface of trophozoite contributes to reduce adherence to galactose and N‐acetylgalactosamine of host cells. Moreover, we also found lower amount of peroxiredoxin (B1N5Y9) (PRX) and superoxide dismutase (SOD) (C4LX20), two enzymes involved in resistance of host oxidative defenses, that are important components of amoebic virulence [44]. The reduced amount of 3‐ketoacyl‐CoA synthase 4 or fatty acid elongase (B1N502) could also have an impact on parasite virulence properties that require an active lipid membrane dynamic [45].

### *EhCFIm25* silencing has an impact on gene expression

The last group of proteins with an altered amount in *EhCFIm25*‐silenced trophozoites can be generally clustered around gene expression and protein modifications, which may explain the proteomic changes described above that promotes parasite death and virulence diminution. Most of these proteins showed a reduced abundance or were not detected, in agreement with the high number of downregulated proteins. They include several ribosomal proteins (C4LU56, B1N384, B1N3R9, B1N2Z3, C4M4I3, C4LTQ5, B1N442), aminoacyl‐tRNA synthetases (C4LWW8, C4LXL0, C4LZN0), elongation factors (C4M7D4, B1N306), and an aminopeptidase (C4M0L4), a protein kinase domain‐containing protein (B1N2E5) and a protein disulfide isomerase (C4MB38). Additionally, two proteins that are important for RNA metabolism were not detected: the ATP‐dependent RNA helicase DDX39 (B1N3F1) whose homologue in humans participates in the regulation of transcription, splicing, and RNA export [46] and the polyadenylate‐binding protein_ putative (C4M6Y2) that interacts with the PAP to enhance polyadenylation at the 3′‐end of mRNA and bind the poly(A) tail to avoid transcript degradation and promotes translation through mRNA circularization and interaction with eIF4G [47]. Altogether, our results demonstrate that the absence of EhCFIm25 had an impact on gene expression and proteome, and as a result, on *E. histolytica* survival and virulence properties, however, the molecular mechanisms involved remain unclear. In our previous work, we showed that EhCFIm25 controls the efficient selection of distal (or downstream) poly(A) sites in *E. histolytica* transcripts and hypothesized that its silencing may have an impact on the 3′‐end mRNA processing and therefore gene expression of the 20 identified genes with two polyadenylation sites [14, 48]. Surprisingly, proteins that are modulated in our proteomics analysis did not correspond to any of these genes, which agrees with the limited impact of alternative polyadenylation on gene expression regulation in this parasite. In humans, CFIm25 is also essential for polyadenylation factors recruitment, pre‐mRNA cleavage, and poly(A) tail synthesis [4, 5, 6, 7]. Therefore, it is possible that changes in protein abundance following *EhCFIm25* silencing may result more from alterations in these events that from an upstream shift in poly(A) site selection; future experiments required to be performed to confirm this assumption. On the other hand, it is important to point out that a proteome does not represent the full set of proteins expressed in a cell, and it is an approximation that depends on the identification of peptides and their amount; moreover, the shotgun analysis presented here has been performed in total protein extracts, which may limit the detection of less abundant proteins. Notably, several genes with two poly(A) sites correspond to nuclear proteins that participate in DNA condensation, DNA binding, translation, splicing, and mRNA binding; similarly, the nuclear EhCFIm25 protein was not identified in any of the three proteomes. The absence of nuclear proteins among the identified proteins is likely related to the lower amount of nuclear proteins in comparison with cytoplasmic proteins. It is possible that a proteomic analysis of nuclear proteins would give some insights about the abundance of these specific proteins. Additionally, the increased expression of EhPAP and EhPC4 that interact with EhCFIm25 suggests the existence of compensatory mechanisms in gene expression regulation that may help to overcome the absence of EhCFIm25 and reduce, at least partially, alterations in transcription and RNA processing, for parasite survival during the first days.

In conclusion, the present proteomic approach confirmed the significance of EhCFIm25 as a biochemical target in *E. histolytica* by providing interesting insights about the proteins and biological pathways that are altered following the absence of this polyadenylation factor. Notably, our results suggested that EhCFIm25 silencing affected energy metabolism and cytoskeleton dynamics causing parasite death and reducing parasite virulence, respectively, by altering gene expression, which explains parasite death, and alteration in virulence properties that were observed in the absence of EhCFIm25. The next step will involve additional studies to confirm the hypotheses emerging from our proteomic analysis and get a comprehensive view of the molecular mechanisms involved.

## Conflict of interest

The authors declare no conflict of interest.

## Author contributions

LAM and CLC conceived and supervised the study; LAM and ERM designed experiments; AISM and RGAB performed experiments and bioinformatics analyses; CACS provided new strategies and reagents; RGAB and ERM analyzed data; LAM, AISM, and RGAB wrote the manuscript; ERM, CLC, and CACS made manuscript revisions.

## Supporting information

**Fig S1.** Effect of EhCFIm25 silencing on *E. histolytica* trophozoites proliferation and viability. Trophozoites (5.0 × 10^4^) were soaked with *EhCFIm25*‐dsRNA (100 μL·mL^−1^) at 37 °C. At day 4, total RNA was obtained to evaluate *EhCFIm25* mRNA expression by Real‐time quantitative reverse transcription polymerase chain reaction (Real‐time qPCR). The *EhRNAPII* gene mRNA expression was determined and used as normalization control. Data corresponding to the *EhCFIm25*‐dsRNA condition were compared to both control conditions using the paired Student's *t*‐test. **P < 0.01; ***P < 0.001 (A). Each day, cells were counted (B) and parasite viability was assessed by the Trypan blue assay (C). Data corresponding to the *EhCFIm25*‐dsRNA condition were compared to both control conditions using the two‐way ANOVA test. *P < 0.05 and ***P < 0.001. *n* = 3. Error bars represent SD.

**Fig S2.** Real‐time quantitative reverse transcription polymerase chain reaction (Real‐time qRT‐PCR) for EhPAP and EhPC4 genes in EhCFIm25‐dsRNA trophozoites compared with (A) non‐treated cells and (B) gfp‐dsRNA treated parasites. The EhRNAPII gene mRNA expression was determined and used as normalization control for all qRT‐PCR assays. Data corresponding to the EhCFIm25‐dsRNA condition were compared to both control conditions using the paired Student's *t*‐test. *P < 0.05; ***P < 0.001. *n* = 3. Error bars represent SD.

**Table S1.** Raw data.

## Data Availability

The data that support the findings of this study are openly available in the ProteomeXchange Consortium at http://www.proteomexchange.org/, reference number PXD027784.
