# Supplementary figures and images for: Unraveling the relevance of the polyadenylation factor EhCFIm25 in Entamoeba histolytica through proteomic analysis
Source: FEBS Open Bio. 2021 Sep 13;11(10):2819–35. doi: 10.1002/2211-5463.13287 (PMC8487052; doi:10.1002/2211-5463.13287)

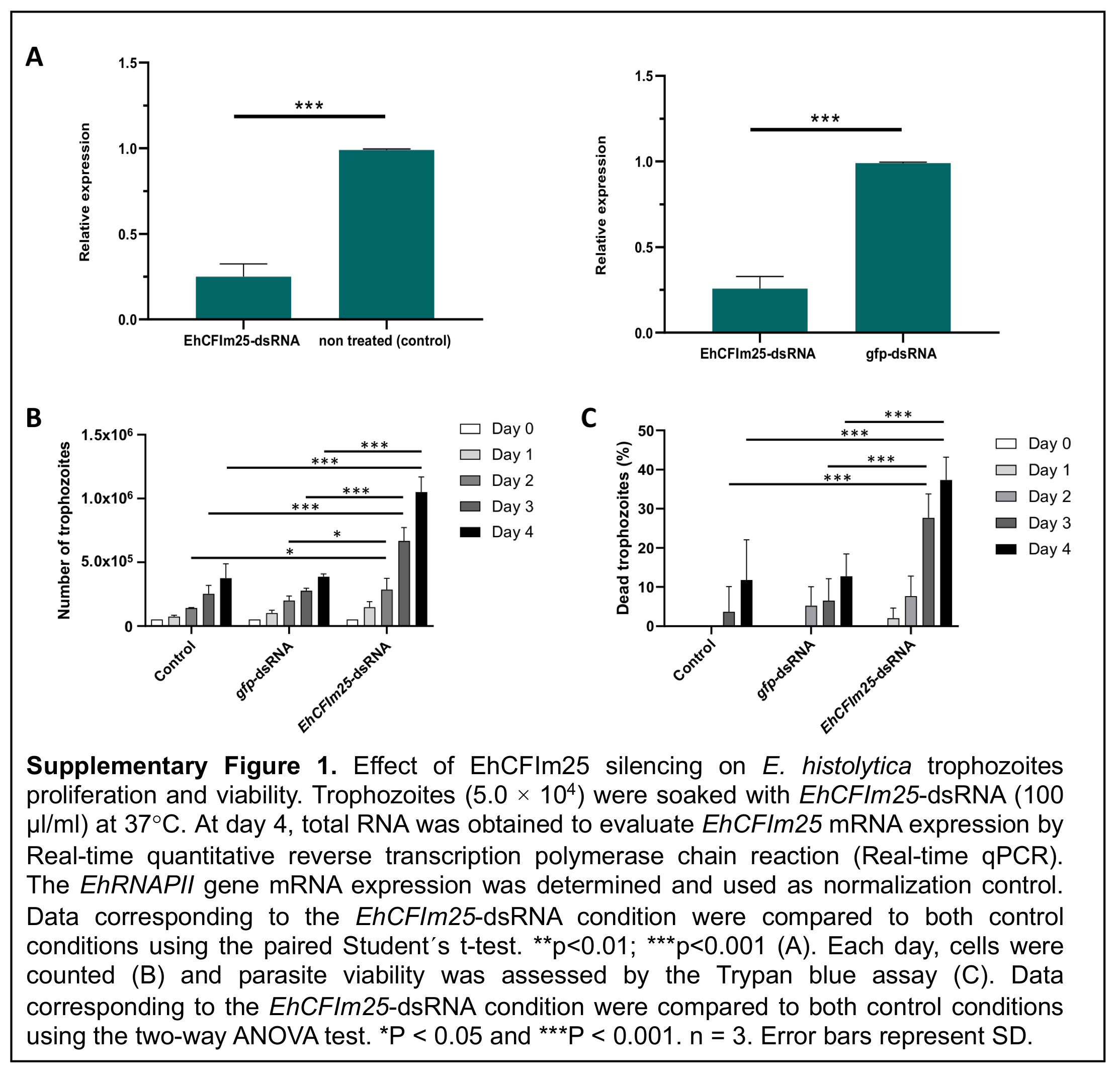

Supplement: Supplementary file 1 — Fig S1. Effect of EhCFIm25 silencing on E. histolytica trophozoites proliferation and viability. Trophozoites (5.0 × 10^4^) were soaked with EhCFIm25‐dsRNA (100 μL·mL−1) at 37 °C. At day 4, total RNA was obtained to evaluate EhCFIm25 mRNA expression by Real‐time quantitative reverse transcription polymerase chain reaction (Real‐time qPCR). The EhRNAPII gene mRNA expression was determined and used as normalization control. Data corresponding to the EhCFIm25‐dsRNA condition were compared to both control conditions using the paired Student's t‐test. **P < 0.01; ***P < 0.001 (A). Each day, cells were counted (B) and parasite viability was assessed by the Trypan blue assay (C). Data corresponding to the EhCFIm25‐dsRNA condition were compared to both control conditions using the two‐way ANOVA test. *P < 0.05 and ***P < 0.001. n = 3. Error bars represent SD. [file FEB4-11-2819-s003.tif]

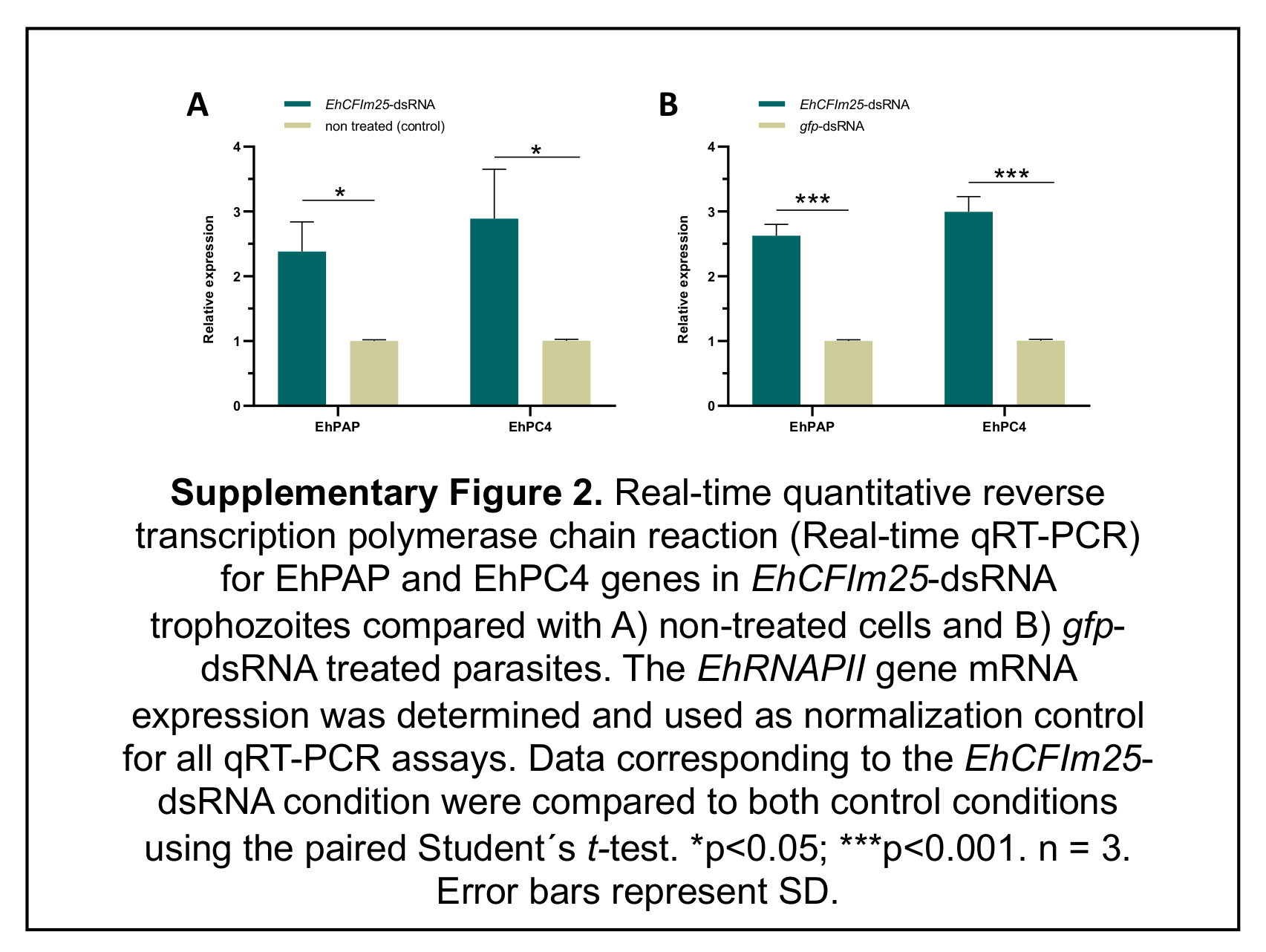

Supplement: Supplementary file 2 — Fig S2. Real‐time quantitative reverse transcription polymerase chain reaction (Real‐time qRT‐PCR) for EhPAP and EhPC4 genes in EhCFIm25‐dsRNA trophozoites compared with (A) non‐treated cells and (B) gfp‐dsRNA treated parasites. The EhRNAPII gene mRNA expression was determined and used as normalization control for all qRT‐PCR assays. Data corresponding to the EhCFIm25‐dsRNA condition were compared to both control conditions using the paired Student's t‐test. *P < 0.05; ***P < 0.001. n = 3. Error bars represent SD. [file FEB4-11-2819-s001.tif]
